# Engagement-Supportive Mechanisms in Stress-Focused Digital Mental Health Interventions With Adaptive, Conversational, and Context-Aware Systems: Narrative Review

**DOI:** 10.2196/90855

**Published:** 2026-11-02

**Authors:** Munesu Maminimini, Sofia Asteria Piffer, Silvia Gabrielli

**Affiliations:** 1 Department of General Psychology, University of Padua, Via Luigi Luzzatti, 4, Padova, Veneto, 35121, Italy, 39 348 610 4125; 2 Center for Digital Health & Wellbeing, Fondazione Bruno Kessler, Trento, Trentino-Alto Adige, Italy

**Keywords:** digital mental health interventions, stress management, user engagement, just-in-time adaptive interventions, conversational agents, artificial intelligence

## Abstract

**Background:**

Stress is a widespread public health concern associated with cardiovascular disease, depression, anxiety, and reduced quality of life. Digital mental health interventions (DMHIs), delivered through smartphones, web platforms, and wearables, offer scalable support for stress management but often struggle to sustain user engagement. Evidence describing how adaptive, conversational, and context-aware mechanisms function together within stress-focused DMHIs remains fragmented.

**Objective:**

This review aims to synthesize evidence on engagement-supportive mechanisms in stress-focused DMHIs, with attention to personalization; biometric feedback; just-in-time adaptive interventions (JITAIs); gamification; AI, including large language models (LLMs); and methodological approaches to studying engagement. It also examines methodological, motivational, and experiential dimensions of engagement.

**Methods:**

This narrative review was informed by a structured search of PubMed, IEEE Xplore, and Scopus for peer-reviewed studies published between January 2018 and April 2025, followed by an evidence update through August 2026. Eligible studies described stress-focused DMHIs or related affective support interventions reporting at least 1 engagement-supportive mechanism. Randomized controlled trials, qualitative investigations, simulation or modeling studies, and implementation studies were included. Data were extracted using a structured form capturing study characteristics, engagement mechanisms, outcomes, and alignment with the review domains. Descriptive mapping summarized study types and mechanism frequencies, while a narrative synthesis integrated behavioral, experiential, methodological, and theory-informed findings.

**Results:**

Thirty-two studies met the inclusion criteria. Randomized or microrandomized controlled trials were the most common design (n=17), followed by qualitative investigations (n=6), design or implementation studies (n=3), mixed methods studies (n=3), simulation or modeling studies (n=2), and a quasi-experimental study (n=1). Dynamic adaptation was identified in 14 studies, conversational support in 10, static tailoring in 9, social or co-design mechanisms in 9, context-aware prompting in 7, gamified reinforcement in 5, biometric-responsive adaptation in 4, and narrative immersion in 2. These mechanisms frequently overlapped within layered adaptive architectures rather than functioning as isolated engagement features. AI-enabled systems included conversational agents, LLM-supported systems, recommender systems, reinforcement learning, and computational modeling approaches. Outcomes included behavioral indicators such as adherence, retention, and prompt receptivity, alongside experiential indicators such as perceived relevance, trust, emotional support, and immersion. Evidence strength varied substantially; many AI-enabled systems contributed feasibility, acceptability, or optimization-oriented evidence rather than definitive comparative effectiveness evidence.

**Conclusions:**

Stress-focused DMHIs are increasingly adopting adaptive, conversational, and context-aware engagement-supportive mechanisms, but the evidence base remains heterogeneous and methodologically uneven. Dynamic adaptation, conversational support, and static tailoring were most frequently represented, whereas biometric-responsive adaptation, narrative immersion, and deeply integrated gamified systems were less common. AI-enabled systems may support contextual responsiveness and personalized interaction but require transparent adaptive logic, privacy safeguards, and human-centered evaluation. Future work should prioritize longitudinal, mixed methods research capable of distinguishing behavioral engagement, experiential engagement, feasibility, acceptability, and sustained stress-related outcomes.

## Introduction

Stress is a pervasive psychological and physiological response to perceived internal or external demands, and when it is prolonged or maladaptively regulated, it may contribute to psychological distress and stress-related disorders including anxiety, depression, sleep disturbance, and poorer quality of life [1,2]. Digital mental health interventions (DMHIs), delivered via mobile apps, web platforms, and wearables, can provide scalable, low-threshold support through psychoeducation, monitoring, and behavioral strategies. Although meta-analyses and recent randomized controlled trial (RCT) evidence indicate that smartphone-based and app-based interventions can reduce stress or anxiety symptoms [1,3], their long-term impact and effectiveness are often limited by poor engagement and high dropout rates [4].

Engagement in DMHIs is a multidimensional construct encompassing behavioral, cognitive, emotional, experiential, and social dimensions [4-7]. Behavioral engagement is commonly reflected through indicators such as uptake, adherence, frequency and duration of use, and retention and completion rates, while cognitive and experiential engagement relate more closely to perceived relevance, motivational investment, emotional connection, immersion, and trust in the intervention experience [2,5]. Social or participatory dimensions may additionally emerge through co-design processes, peer interaction, clinician support, or blended care approaches [8,9]. Engagement-supportive mechanisms refer to the intervention design or delivery processes intended to influence 1 or more of these dimensions.

The “law of attrition” describes the declining DMHI use and loss to follow-up over time [4]. Barriers including low perceived relevance, burdensome data entry, and inadequate feedback may contribute to disengagement [2]. Reviews have consequently emphasized user-centered design and adaptive technologies as possible means of supporting continued engagement [5,10]. To improve user engagement and retention, recent DMHI research has explored personalization, biometric feedback, gamification, just-in-time adaptive interventions (JITAIs), and AI-enabled conversational or recommendation systems.

Personalization may use demographic characteristics, preferences, psychological profiles, or behavioral data to increase perceived relevance and autonomy [11-14]. Biometric and ecological momentary assessment (EMA) data can inform JITAIs that seek to provide support when it is needed and users are receptive [2,15,16]. Gamification uses elements such as challenges, feedback, points, or narratives to encourage persistence, although poorly aligned elements may be ineffective or counterproductive [17-20]. Conversational and other AI-enabled systems may provide responsive psychoeducation and support at scale while raising concerns about safety, privacy, transparency, and perceived authenticity [21-27]. Together, these approaches address the relevance, timing, and experience of digital support and may overlap within the same intervention.

Although individual mechanisms have been examined, evidence describing how they are implemented, combined, and assessed in stress-focused DMHIs remains fragmented. Previous work has mapped broad engagement barriers [2,6,28], examined personalization, or JITAI elements [5,29-31] and examined chatbots, gamification, and recommender systems [11,17,18,21,32]. Recent syntheses continue to identify inconsistent engagement definitions, heterogeneous measures, and limited longitudinal evidence [33-36]. What remains less clear is how several mechanisms operate together; how static tailoring differs from dynamic adaptivity; and what controlled, qualitative, implementation, and computational studies each contribute to understanding engagement.

Accordingly, this narrative review aims to synthesize and critically evaluate how stress-focused DMHIs conceptualize and operationalize engagement; how personalization, biometric feedback, JITAI timing, gamification, and AI-enabled approaches are used to support it; and how different methodological approaches contribute to understanding their implementation and effects.

## Methods

### Design

This narrative review was informed by structured systematic search, screening, and extraction procedures. A narrative synthesis was selected because the evidence spans clinical trials, qualitative inquiry, implementation research, and computational studies and because the review examines how engagement mechanisms are implemented and interpreted rather than estimating a pooled effect. A completed PRISMA-P (Preferred Reporting Items for Systematic Review and Meta-Analysis Protocols) checklist is provided in Checklist 1.

### Search Strategy

The original search covered PubMed, IEEE Xplore, and Scopus from January 2018 through April 2025. Search concepts combined digital mental health, stress or distress, engagement, and adaptive mechanisms, including personalization, biometric feedback, EMA, JITAIs, gamification, conversational agents, LLMs, and reinforcement learning. Well-being terminology was also considered because preventative DMHIs may describe emotional support or coping without using stress reduction as their primary label. A further update covering May 2025 through August 2026 was conducted using the same concept blocks for the focused database searches and a supplementary journal search of recent peer-reviewed work. The review domains and illustrative search concepts are summarized in Table 1, and the full database-specific strings, dates, and the supplementary search description are reported in Multimedia Appendix 1.

**Table 1.** Review domains and illustrative search concepts.

Review domain	Search query
Engagement-supportive mechanisms and behavioral or experiential engagement outcomes	(“digital health” OR “mHealth” OR “eHealth”) AND (“engagement” OR “adherence” OR “retention”) AND (“stress” OR “chronic diseases”)
Personalization, biometric feedback, JITAI^a^ timing, and gamification	(“personalisation” OR “tailoring” OR “adaptive intervention” OR “biometric data” OR “heart rate” OR “EMA” OR “ecological momentary assessment” OR “JITAI” OR “just-in-time” OR “gamification”) AND (“engagement” OR “adherence” OR “retention”) AND (“digital health” OR “mHealth” OR “eHealth”) AND (“stress”)
AI-enabled adaptive and engagement-oriented personalization	(“artificial intelligence” OR “machine learning” OR “adaptive systems” OR “LLM” OR “large language models”) AND (“digital health” OR “personalised intervention”)
Methodological contributions to engagement-informed DMHI^b^ design	(“self-research” OR “autoethnography”) AND (“digital health” OR “engagement”)

a JITAI: just-in-time adaptive intervention.

b DMHI: digital mental health intervention.

### Eligibility Criteria

Studies were included if they (1) described a DMHI aiming primarily to reduce stress or improve related affective outcomes within preventative mental health contexts; (2) reported at least 1 engagement-supportive mechanism, such as personalization, biometric feedback, JITAI, gamification, or AI or chatbot functionality, implicitly or explicitly; (3) presented empirical, qualitative, implementation, simulation, modeling, or system design evidence relevant to engagement; (4) were peer-reviewed; and (5) were published in English. Protocols, reviews, dissertations, nondigital interventions, and non–mental health interventions were excluded. A study was also eligible if it addressed 1 or more engagement-supportive mechanisms and met the broader stress-focused criteria; it did not need to include every mechanism or use the term “engagement” explicitly. Commercial applications, app store–only evaluations, and other gray literature were out of scope unless evaluated in a peer-reviewed study meeting these criteria. Two technical preprints from the original corpus were retained as a narrow exception because they addressed emerging AI and reinforcement learning architectures [25,37]; they were interpreted as design or computational evidence rather than clinical effectiveness evidence. No new preprints were added, and the evidence update did not broaden the eligibility criteria.

### Study Selection and Data Extraction

MM and SAP independently screened titles and abstracts and then assessed potentially eligible full texts in the original review. Disagreements were resolved through discussion with SG until consensus was reached. MM and SAP extracted data from studies identified in the original search using the structured 17-item form described below. For studies identified in the search update, MM conducted the initial screening and data extraction using the unchanged criteria, and SG verified the draft eligibility decisions, extracted data, and interpretations.

A structured 17-item form captured publication details, population, setting, intervention modality, duration, follow-up, study design, engagement definitions and measures, engagement-supportive mechanisms, AI or sensing components, key findings, and design implications. The same form was applied to the papers identified in the search update. Eligibility and mechanism coding were kept separate so that methodological studies could be included in the corpus without being misclassified as intervention-level engagement mechanisms.

### Synthesis Approach

The synthesis proceeded in 2 stages. First, descriptive mapping characterized study design, population, modality, duration, and follow-up. Second, overlapping mechanisms were coded as static tailoring, dynamic adaptation, conversational support, context-aware prompting, biometric-responsive adaptation, gamified reinforcement, narrative immersion, or social and co-design support. In this review, AI encompassed conversational agents and LLM-based systems; recommender systems; contextual bandits; reinforcement learning, rule-based, or fuzzy logic approaches; and simulation or modeling used for assessment, timing, content selection, or interaction. Higher-level labels such as JITAI or AI were classified according to their functional role in sensing, decision-making, or delivery.

Findings were synthesized across behavioral, cognitive, experiential, relational, and methodological evidence. Behavioral indicators were distinguished from subjective or interaction-oriented engagement processes, and associations were interpreted descriptively because interventions frequently combined mechanisms and rarely isolated their independent effects.

Self-determination theory (SDT) informed the interpretation of motivational and experiential engagement. Meaningful choice may support autonomy, appropriate feedback and achievable challenges may support competence, and socially responsive interaction may support relatedness [38,39]. Work on immersive digital environments provided further context on need satisfaction, motivation, enjoyment, and continued participation [40]. These were theory-informed interpretations and included studies were not assumed to have explicitly adopted or tested SDT.

### Ethical Considerations

This review analyzed published reports only and did not recruit participants, intervene in care, or collect or process identifiable personal data. Ethics approval was therefore not obtained. This decision was consistent with the University of Padua ethics self-assessment and JMIR guidance [41,42]. Informed consent and participant compensation were not applicable.

## Results

### Study Selection

Figure 1 summarizes the selection process. The original search identified 2088 records; 179 duplicates were removed, and 1909 records were screened. After 1797 title or abstract exclusions, 112 full-text reports were assessed, and 22 studies met the original criteria. Eleven unique peer-reviewed candidate empirical papers identified during the evidence update were screened against the same criteria [43-53]. Ten studies met all criteria and were included in the review corpus [43-48,50-53]. The insomnia component trial [49] fell outside the stress or related affective eligibility boundary and was retained only as external interpretive evidence. Four reviews and meta-analyses [33-36] also informed interpretation but were not counted. The final review corpus comprised 32 studies.

**Figure 1.**
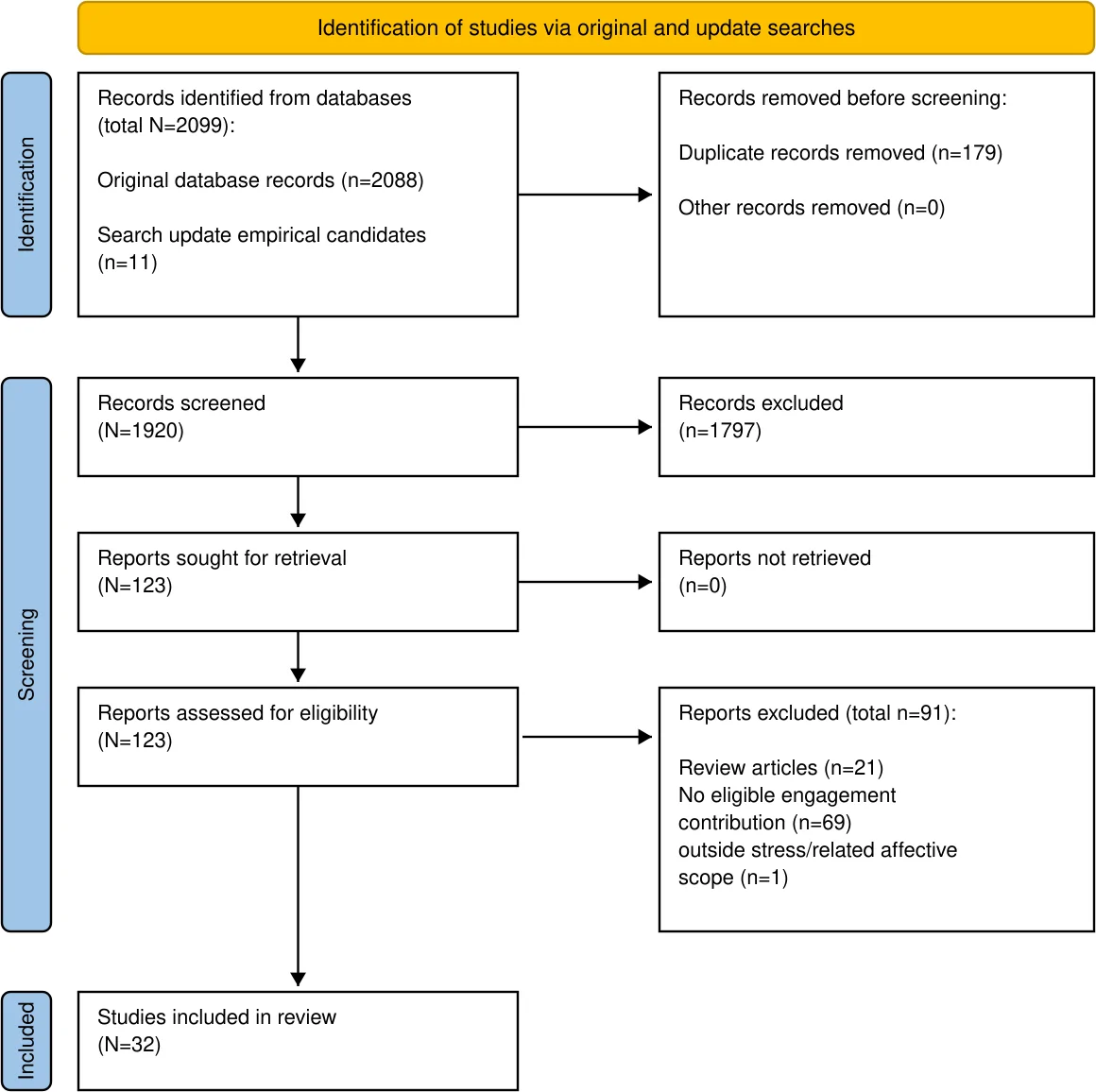
Study selection across the original and update searches. Original database search covered January 2018 to April 2025 (n=2088), and an update covering May 2025 to August 2026 was conducted (11 unique, peer-reviewed empirical candidates). Four reviews and meta-analyses used only as contextual evidence were not counted in this empirical selection flow.

### Overview of Included Studies

The 32 included studies were published between 2021 and 2026 [8,9,12-14,24-27,37,43-48,50-65]. Of these, 30 appeared from 2024 onward, while 1 was published in 2021 [59] and 1 in 2023 [65], reflecting the rapid recent growth of adaptive, context-aware, and AI-enabled DMHI research. Dissemination spanned clinical, digital health, human-computer interaction (HCI), and computing journals and conferences, illustrating the multidisciplinary intersection of mental health, behavioral science, and technology.

Most empirical studies were conducted in high-income settings in North America, Western Europe, Australia, New Zealand, East Asia, and Israel. Culturally adapted interventions in Jordan provided evidence from an otherwise underrepresented regional context [57,63]. Target populations included adolescents, young adults, and students with stress-, anxiety-, or mood-related needs; caregivers and parents; employees and health care trainees exposed to occupational stress; people awaiting psychotherapy; users of youth mental health platforms or mental health chatbots; breast cancer survivors; and people with chronic physical illnesses, including chronic obstructive pulmonary disease [8,9,12-14,24-27,37,43-48,50-65].

Intervention modalities were correspondingly diverse. They included mobile apps with monitoring or guided coping strategies [13,14,43]; web-based and blended programs [8,12,47,52]; wearable-integrated systems using physiological or stress detection to trigger support [37,56]; narrative or gamified interventions [51,55,59,65]; chatbot- and LLM-based conversational systems [24-27,46,53,61,63]; context-aware JITAIs [37,43,44,48,56,64,65]; and simulation, modeling, or adaptive trial frameworks relevant to decision-making and optimization [45,50,52,54,62]. Across these modalities, engagement was examined through behavioral use and adherence, perceived relevance and burden, emotional or relational experience, contextual receptivity, and methodological optimization. Modalities and engagement-supportive mechanisms frequently overlapped within the same intervention.

Study characteristics and design distribution are summarized in Table 2. Randomized or microrandomized controlled trials were the most common primary design, accounting for 53% (17/32) of the studies [12,13,24,26,44-46,48,50-52,57,59,61,63-65]. Qualitative investigations accounted for 19% (6/32), including interview-based, experiential, diary-based, and reflective designs [9,14,27,53,56,58]. Design or implementation studies accounted for 9% (3/32) [8,25,37]. Mixed methods studies also accounted for 9% (3/32) [47,55,60]. Simulation or modeling studies represented 6% (2/32) [54,62], while 1 quasi-experimental study accounted for 3% (1/32) [43]. These mutually exclusive categories reflect each study’s primary methodological contribution, as some controlled trials also incorporated mixed methods components. Overall, 81% (26/32) of the included papers reported empirical studies involving conventional participant samples [8,12-14,24,26,27,43-48,50-53,55,57-61,63-65], while 19% (6/32) focused on design, reflexive inquiry, simulation, modeling, or reinforcement learning approaches without a conventional participant sample [9,25,37,54,56,62].

**Table 2.** General characteristics of included studies.

Study	Title	Study type	Duration	Follow-up
Weintraub et al [12]	Effects of a multifamily app-enhanced cognitive-behavioral therapy delivered via telehealth for parents of adolescents with mood or psychotic spectrum disorders	RCT^a^; mixed methods	9-week treatment; 3-month posttreatment follow-up	Fᵖ^b^
Sirbu et al [13]	Comparative efficacy of PsyPills and OCAT mobile psychological interventions in reducing depressive, anxiety and stress symptoms: a blinded randomized clinical trial	Blinded RCT	10-day intervention; 1-month follow-up	Fᵖ
Wang et al [54]	HealthQ: unveiling questioning capabilities of LLM^c^ chains in healthcare conversations	Simulation study	N/A^d^	N/A
Heinz et al [24]	Randomized trial of a generative AI chatbot for mental health treatment	RCT	4-week Therabot intervention; follow-up at 8 weeks	Fᵖ
Guo et al [25]	SouLLMate: an adaptive LLM-driven system for advanced mental health support and assessment, based on a systematic application survey	Survey and design	N/A	N/A
Sadprasid et al [55]	Leveraging idle games to incentivize intermittent and frequent practice of deep breathing	Mixed methods	4-week at-home deployment; 2 weeks per app condition	N/A
Neupane et al [56]	Wearable meets LLM for stress management: a duoethnographic study integrating wearable-triggered stressors and LLM chatbots for personalized interventions	Duoethnography study	22-day duoethnographic use period	N/A
Alvarez-Jimenez et al [8]	A national evaluation of a multi-modal, blended, digital intervention integrated within Australian youth mental health services	Implementation study	32-month implementation; 12-week clinical support allocation; engagement tracked to 24 weeks	Fᵖ
Miller et al [9]	Facilitating co-design in healthcare: a collaborative autoethnography of personalities, power, and competing priorities	Autoethnography design	N/A	N/A
Dardas et al [57]	Validating a digital depression prevention program for adolescents in Jordan: cultural adaptation and user testing in a randomized controlled trial	RCT; mixed methods	10-session/module intervention; exact intervention duration not clearly reported	N/A
Rutherford et al [58]	Experiences of home‐based pulmonary rehabilitation with mHealth and ‐based pulmonary rehabilitation in people with chronic obstructive pulmonary disease: a qualitative study	Qualitative study	8-week pulmonary rehabilitation program	N/A
Schwarz et al [59]	Does dynamic tailoring of a narrative-driven exergame result in higher user engagement among adolescents? Results from a cluster-randomized controlled trial	Cluster RCT	4-week game-based intervention period	N/A
Slade et al [60]	Design guidelines for improving mobile sensing data collection: prospective mixed methods study	Mixed methods	30-day mobile sensing user study	Poststudy/exit survey only^e^
Valentine et al [14]	A personalized, transdiagnostic smartphone app (Mello) targeting repetitive negative thinking for depression and anxiety: qualitative analysis of young people’s experience	Qualitative study	6-week Mello intervention period	Fᵖ
Campellone et al [26]	Safety and user experience of a generative artificial intelligence digital mental health intervention: exploratory randomized controlled trial	Exploratory RCT	2-week exploratory randomized trial	N/A
Tong et al [61]	Effectiveness of topic-based chatbots on mental health self-care and mental well-being: randomized controlled trial	RCT	10-day chatbot intervention; 1-month follow-up	Fᵖ
Ying et al [62]	Mental health assessment model for college students using circular q-rung orthopair fuzzy Muirhead means and MULTIMOORA method	Computational modeling	N/A	N/A
de Graaff et al [63]	Evaluation of a guided chatbot intervention for young people in Jordan: feasibility randomized controlled trial	Feasibility RCT	8-week STARS^f^ chatbot intervention	N/A
Coppens et al [64]	Exploring the added effect of three recommender system techniques in mobile health interventions for physical activity: a longitudinal randomized controlled trial	Longitudinal RCT	8-week app use; 6-month follow-up questionnaire	Fᵖ
Siddals et al [27]	“It happened to be the perfect thing”: experiences of generative AI chatbots for mental health	Qualitative study	N/A	N/A
Wang and Fang [65]	Effectiveness of gamified team competition as mHealth intervention for medical interns: a cluster micro-randomized trial	Micro-RCT	≈12-week intervention	N/A
Aqajari et al [37]	Enhancing performance and user engagement in everyday stress monitoring: a context-aware active reinforcement learning approach	Reinforcement learning study	N/A	N/A
Kor et al [43]	Developing and testing a brief mindfulness just-in-time adaptive intervention to reduce stress among caregivers of people with dementia: quasi-experimental study	Quasi-experimental study	18-day intervention	N/A
Elmer et al [44]	A social support just-in-time adaptive intervention for individuals with depressive symptoms: feasibility study with a microrandomized trial design	Feasibility micro-RCT, mixed methods	18-day intervention	N/A
Newby et al [45]	Brief digital interventions for psychological distress: an AI-enhanced response-adaptive randomized clinical trial	Response-adaptive RCT	2-week onboarding plus 2-week intervention	N/A
Shoshani et al [46]	Efficacy of a conversational AI agent for psychiatric symptoms and digital therapeutic alliance: a randomized clinical trial	3-arm RCT	12-week intervention; 3-month follow-up	Fᵖ
Valentine et al [47]	Sustained engagement with a digital youth mental health platform: a mixed-methods study	Convergent mixed methods study	Real-world platform use; no conventional fixed duration reported	N/A
Dai et al [48]	A design framework for context-aware digital micro-interventions: integrating emotion recognition and just-in-time adaptive intervention for workplace stress management	Design framework and 2-arm RCT	6-week RCT	N/A
Chow et al [50]	Digital mental health treatment and symptoms of depression and anxiety in breast cancer survivors: a randomized clinical trial	Sequential multiple assignment randomized trial	8-week intervention; 6- and 12-month follow-up	Fᵖ
Barry et al [51]	A digital behavioral health game for adolescent mental health: a prespecified secondary analysis of a randomized clinical trial	Prespecified secondary analysis of an RCT	6-week intervention; 12-month follow-up	Fᵖ
Rouvere et al [52]	Personalization strategies for increasing engagement with digital mental health resources: sequential multiple assignment randomized trial	Sequential multiple assignment randomized trial	Single-session real-world website experiment	N/A
Xu et al [53]	The digital therapeutic alliance with mental health chatbots: diary study and thematic analysis	Longitudinal diary study and reflexive thematic analysis	4-week user study	N/A

a RCT: randomized controlled trial.

b Fᵖ refers to an assessment or engagement observation beyond the immediate intervention or primary postassessment period.

c LLM: large language model.

d N/A denotes studies where duration or follow-up could not be meaningfully defined, such as simulation, modeling, design, or autoethnographic studies, or where no follow-up was reported.

e “Poststudy/exit survey only” indicates assessment at the end of the study period rather than longer-term follow-up.

f STARS: Scalable Technology for Adolescents and Youth to Reduce Stress.

### Engagement-Supportive Mechanisms

Table 3 summarizes the presence or absence of the primary engagement-supportive mechanisms identified across the 32 included studies. Studies were classified using overlapping mechanism categories rather than mutually exclusive intervention types because many stress-focused DMHIs combined adaptive, conversational, contextual, motivational, or social components within the same intervention architecture.

Personalization reflected 2 conceptually distinct approaches. Static tailoring appeared in 28% (9/32) of the studies and used predefined characteristics, preferences, symptom profiles, cultural factors, or user-provided information to select or modify support [8,12,13,43,44,52,57,63,64]. Dynamic adaptation appeared in 44% (14/32) and used accumulating behavioral, contextual, physiological, or conversational information to modify intervention timing, content, allocation, or interaction over time [14,24-26,37,43,44,46,48,50,54,56,59,64]. Some interventions combined both approaches [43,44,64], illustrating that static and dynamic personalization are related but not interchangeable. Personalization did not uniformly improve engagement. In Rouvere et al [52], additional tailoring questions increased disengagement, while the effects of tailoring differed according to the information used.

Conversational support appeared in 31% (10/32) of the studies [24-27,46,53,54,56,61,63]. These interventions used chatbot-based or guided dialogue to provide psychoeducation, emotional responsiveness, reflective interaction, or contextually relevant support. Conversational studies reported acceptability and user experience outcomes as well as relational dimensions such as therapeutic alliance and perceived bonding. Shoshani et al [46] found that perceived therapeutic alliance was associated with engagement and symptom improvement, while Xu et al [53] identified conversational control; input fit; caring responses; useful advice; colloquial language; and a private, nonjudgmental setting as factors shaping users’ bonds with mental health chatbots.

Social or co-design mechanisms appeared in 28% (9/32) of the studies [8,9,44,47,50,51,58,63,65]. These included participatory design, blended care, culturally responsive guidance, peer or team interaction, support network activation, and selective human coaching. Valentine et al [47] associated sustained engagement with a youth mental health platform with validation, normalization, belonging, and the practical application of therapeutic insights. Chow et al [50] reported that adding human coaching increased the number of days of app use among participants whose use was low during the first intervention week.

Context-aware prompting appeared in 22% (7/32) of the studies [14,37,43,44,48,56,64]. These systems adjusted intervention delivery according to behavioral, contextual, physiological, or user-reported information. Receptivity and intervention timing were examined directly in several context-aware studies. Kor et al [43] used accumulating receptivity data to inform mindfulness prompt delivery, Elmer et al [44] found that prompts triggered by a stated need for support were rated as better timed and more helpful than prompts triggered by distress thresholds, and Dai et al [48] reported higher adherence with context-aware delivery than fixed delivery.

Biometric-responsive adaptation appeared in 13% (4/32) of the studies [37,48,56,59]. Physiological or emotion recognition data generally functioned as inputs to contextual assessment, prompting, or adaptive delivery rather than as independent engagement mechanisms. Gamified reinforcement appeared in 16% (5/32) of the studies [12,51,55,59,65], while narrative immersion appeared in 6% (2/32) of the studies [51,59]. Their implementation and findings varied: an idle breathing game increased practice frequency [55]; dynamic tailoring increased narrative presence without improving measured immersion, while play time and completed sessions were lower in the tailored condition [59]; and team competition produced early affective benefits that weakened over time [65]. In Barry et al [51], a co-designed narrative game reduced depressive symptoms relative to an active control over 12 months but did not improve anxiety; mechanism-specific engagement or narrative resonance outcomes were not reported.

These frequencies should be interpreted cautiously. The review intentionally foregrounded adaptive, technologically mediated, and engagement-supportive interventions; therefore, the distribution within this selected corpus should not be treated as an estimate of mechanism prevalence across all DMHIs. In addition, 3 studies [45,60,62] were not assigned a mechanism (Table 3). Slade et al [60] examined sensing compliance, user control, and data collection burden; Ying et al [62] contributed a computational mental health assessment model; and Newby et al [45] used response-adaptive allocation across trial participants rather than an intervention-level engagement mechanism. These studies remained eligible because they contributed methodological or computational evidence relevant to engagement and adaptive intervention design.

**Table 3.** Presence (✓) or absence (—) of engagement-supportive mechanisms in each included study.^a^

Study	Static tailoring	Dynamic adaptation	Conversational support	Context-aware prompting	Biometric-responsive adaptation	Gamified reinforcement	Narrative immersion	Social/co-design
Weintraub et al [12]	✓	—	—	—	—	✓	—	—
Sirbu et al [13]	✓	—	—	—	—	—	—	—
Wang et al [54]	—	✓	✓	—	—	—	—	—
Heinz et al [24]	—	✓	✓	—	—	—	—	—
Guo et al [25]	—	✓	✓	—	—	—	—	—
Sadprasid et al [55]	—	—	—	—	—	✓	—	—
Neupane et al [56]	—	✓	✓	✓	✓	—	—	—
Alvarez‑Jimenez et al [8]	✓	—	—	—	—	—	—	✓
Miller et al [9]	—	—	—	—	—	—	—	✓
Dardas et al [57]	✓	—	—	—	—	—	—	—
Rutherford et al [58]	—	—	—	—	—	—	—	✓
Schwarz et al [59]	—	✓	—	—	✓	✓	✓	—
Slade et al [60]	—	—	—	—	—	—	—	—
Valentine et al [14]	—	✓	—	✓	—	—	—	—
Campellone et al [26]	—	✓	✓	—	—	—	—	—
Tong et al [61]	—	—	✓	—	—	—	—	—
Ying et al [62]	—	—	—	—	—	—	—	—
de Graaff et al [63]	✓	—	✓	—	—	—	—	✓
Coppens et al [64]	✓	✓	—	✓	—	—	—	—
Siddals et al [27]	—	—	✓	—	—	—	—	—
Wang (Jitao) et al [65]	—	—	—	—	—	✓	—	✓
Aqajari et al [37]	—	✓	—	✓	✓	—	—	—
Kor et al [43]	✓	✓	—	✓	—	—	—	—
Elmer et al [44]	✓	✓	—	✓	—	—	—	✓
Newby et al [45]	—	—	—	—	—	—	—	—
Shoshani et al [46]	—	✓	✓	—	—	—	—	—
Valentine et al [47]	—	—	—	—	—	—	—	✓
Dai et al [48]	—	✓	—	✓	✓	—	—	—
Chow et al [50]	—	✓	—	—	—	—	—	✓
Barry et al [51]	—	—	—	—	—	✓	✓	✓
Rouvere et al [52]	✓	—	—	—	—	—	—	—
Xu et al [53]	—	—	✓	—	—	—	—	—

a Since many interventions combined adaptive and interdependent mechanisms, the categories presented in this table should be interpreted as overlapping analytical dimensions rather than mutually exclusive intervention types.

### Co-Occurrence of Mechanisms

Figure 2 illustrates the co-occurrence of engagement-supportive mechanisms across the included studies. Dynamic adaptation most frequently co-occurred with context-aware prompting in 7 studies [14,37,43,44,48,56,64] and with conversational support in 6 studies [24-26,46,54,56]. These combinations reflected systems in which behavioral, contextual, or conversational information informed decisions about the timing, content, or form of support.

Biometric-responsive adaptation co-occurred with dynamic delivery in all 4 studies in which it appeared [37,48,56,59] and with context-aware prompting in 3 studies [37,48,56]. This pattern reflects the role of physiological or emotion recognition data as sensing inputs within broader adaptive systems. Context-aware prompting similarly tended to operate alongside personalization, recommendation logic, conversational support, or reinforcement learning rather than as an isolated feature.

Gamified and narrative mechanisms remained comparatively separate from AI-enabled conversational architectures, although Schwarz et al [59] combined dynamic narrative tailoring, biometric responsiveness, and gamified reinforcement. Social mechanisms spanned co-design, blended care, culturally guided support, team competition, support network activation, human coaching, and relational experiences of validation or belonging [8,9,44,47,50,51,58,63,65]. Overall, the co-occurrence patterns show that engagement support was commonly implemented through interacting sensing, decision, and delivery components. These descriptive relationships do not establish that combining more mechanisms necessarily produced stronger engagement.

**Figure 2.**
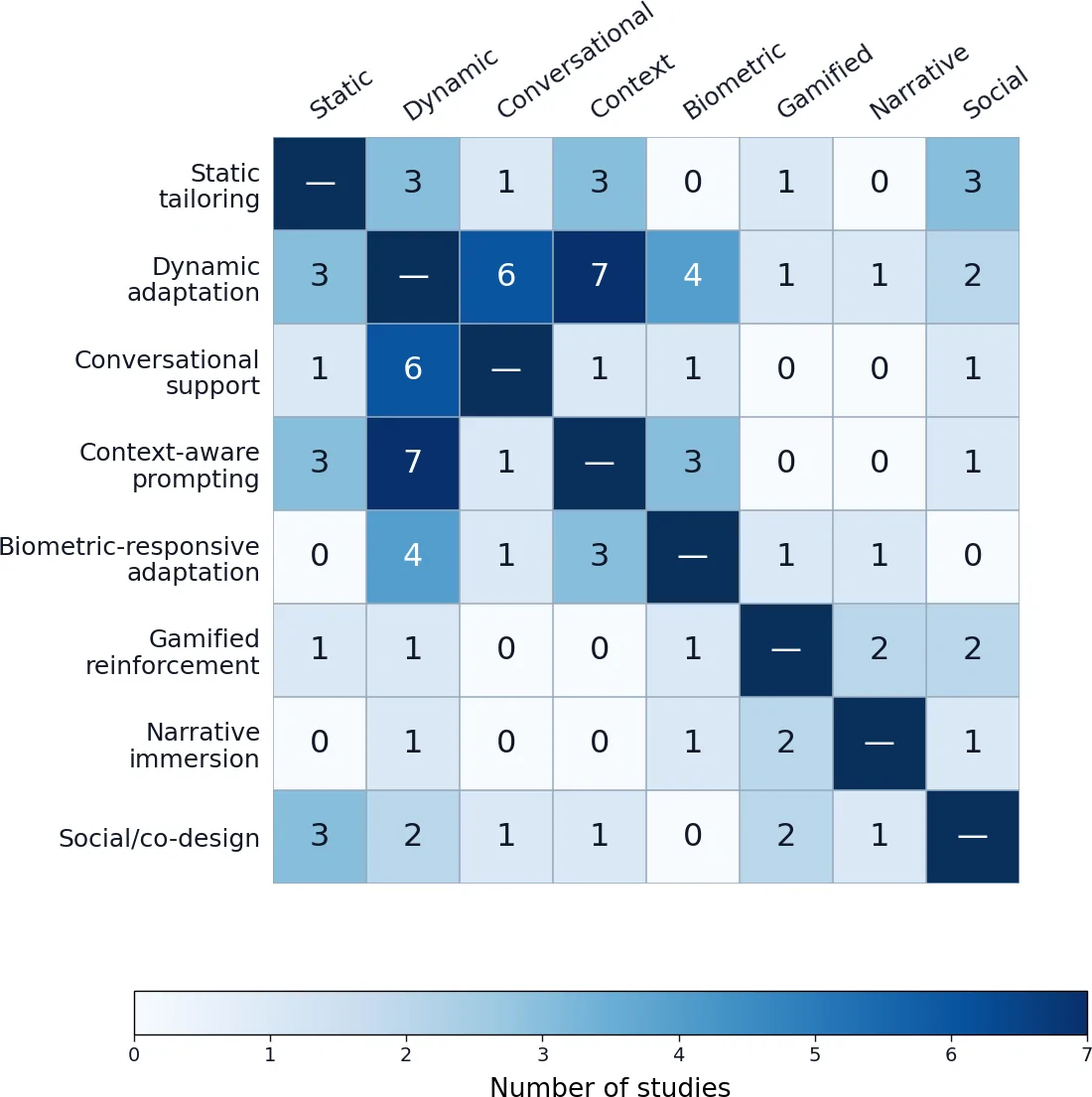
Co-occurrence of engagement strategies across the studies. Values show the number of studies in which each pair of mechanisms co-occurred. Mechanisms are overlapping analytical dimensions and not mutually exclusive intervention categories. Biometric: biometric-responsive adaptation; Context: context-aware prompting; Conversational: conversational support; Dynamic: dynamic adaptation; Gamified: gamified reinforcement; Narrative: narrative immersion; Social: social/co-design; Static: static tailoring.

### AI, Adaptivity, and Methodological Contribution

AI-enabled systems were represented across controlled trials, qualitative studies, simulation and modeling research, and adaptive system design. AI served several distinguishable functions: direct conversational interaction [24-27,46,53,61,63]; within-person adaptation of timing, content, or prompting [37,43,44,48,56,64]; response-adaptive allocation across trial participants [45]; and back-end assessment, simulation, or modeling [25,37,54,62]. Therefore, AI functioned both as an intervention component and as methodological infrastructure for developing or evaluating adaptive systems.

This distinction was particularly important for Newby et al [45]. Its contextual bandit modified allocation probabilities across sequential participants according to distress severity, while the brief interventions received by individual participants remained standardized. The study consequently contributed evidence about AI-enhanced trial methodology rather than dynamically personalized intervention delivery. By contrast, within-person adaptive studies used user-level information to change when or how support was delivered [37,43,44,48,56,64].

The type of evidence also differed across AI implementations. Controlled conversational studies primarily reported completion, acceptability, therapeutic alliance, safety, user experience, or symptom-related outcomes [24,26,46,61,63]. Qualitative and diary studies examined emotional responsiveness, trust, perceived support, conversational fit, and concerns about accuracy or authenticity [27,53,56]. Simulation, modeling, and reinforcement learning studies examined prompt construction, adaptive assessment, recommendation logic, sensing burden, or optimization before or alongside real-world deployment [25,37,45,54,62].

Across the corpus, AI and algorithmic adaptation served distinguishable conversational, within-person adaptive, response-adaptive, and modeling functions. Shoshani et al [46] connected conversational alliance with engagement; Dai et al [48] compared context-aware delivery with fixed delivery; and Newby et al [45] demonstrated the methodological distinction between adapting trial allocation and adapting intervention delivery. Nevertheless, most studies evaluated AI as part of a multicomponent system, and few isolated its independent contribution to sustained engagement.

### Patterns in Engagement Feature Adoption by Methodology

Patterns of mechanism adoption varied across study designs. Static tailoring was prominent in controlled, culturally adapted, and real-world personalization studies [12,13,43,44,52,57,63,64]. Dynamic adaptation appeared more often in AI-enabled, recommender-based, sensing-supported, or contextually responsive systems [14,24-26,37,43,44,46,48,50,54,56,59,64]. Context-aware prompting was concentrated in JITAI, recommendation, and reinforcement learning studies [14,37,43,44,48,56,64], while biometric-responsive adaptation remained limited to sensing-enabled adaptive interventions [37,48,56,59].

Conversational support was represented in both controlled chatbot evaluations and qualitative studies of relational experience [24-27,46,53,54,56,61,63]. Gamified reinforcement appeared primarily in controlled or mixed methods evaluations [12,51,55,59,65], whereas social and co-design mechanisms were particularly visible in qualitative, participatory, implementation, and adaptive support studies [8,9,44,47,50,51,58,63,65].

These methodological patterns affected what could be learned about engagement. Controlled and microrandomized studies contributed behavioral indicators, receptivity data, and symptom-related outcomes; qualitative, diary-based, participatory, and reflective studies revealed trust, burden, cultural fit, power dynamics, and relational experience; and implementation, simulation, and modeling studies clarified scalability, decision logic, and optimization. The resulting evidence types were complementary but not directly interchangeable.

### Study Durations and Follow-Up

Intervention and observation periods varied substantially. Thirteen studies involved intervention or user study periods of approximately 4 weeks or less [13,24,26,43-45,52,53,55,56,59-61]. These included brief app and chatbot trials, a 4-week exergame, a 4-week gamified breathing deployment, an 18-day JITAI, a 22-day wearable LLM study, a 30-day sensing study, and a single-session personalization experiment. Seven studies lasted between 5 and 8 weeks [14,48,50,51,58,63,64], and 3 studies involved 9- to 12-week intervention or support periods [12,46,65]. Alvarez-Jimenez et al [8] was embedded in a longer implementation and tracked engagement for up to 24 weeks.

Eight studies did not report a conventional fixed duration or involved simulation, system design, computational modeling, autoethnography, qualitative accounts of previous use, or real-world platform engagement [9,25,27,37,47,54,57,62]. These studies should not be interpreted as fixed-duration interventions.

Follow-up or assessment beyond the immediate intervention or primary postassessment period was reported in 31% (10/32) of the studies [8,12-14,24,46,50,51,61,64]. Follow-up periods included 1 month [13,61], 8 weeks after a 4-week intervention [24], approximately 3 months [12,46], 6 months [64], engagement tracking for up to 24 weeks [8], and 6- or 12-month assessments in studies of adaptive coaching and narrative game-based support [50,51]. Most studies therefore provided limited evidence about sustained engagement beyond the immediate intervention period.

### Structured Comparison of Findings

Tables 4 and 5 provide complementary summaries of the engagement-supportive mechanisms, AI-enabled systems, methodological approaches, and key findings represented across the included studies. Table 4 presents the engagement-supportive mechanisms identified in each study, the associated findings, and the resulting design implications. The mechanisms represent intervention characteristics, interaction processes, or adaptive functions intended to support engagement; they should not be treated as engagement outcomes themselves. Reported outcomes included behavioral indicators such as uptake, adherence, completion, retention, and receptivity; experiential and relational dimensions such as relevance, burden, immersion, trust, alliance, and emotional support; and qualitative or methodological findings relevant to intervention design.

Across Table 4, controlled studies most commonly contributed behavioral and symptom-related findings, while qualitative and participatory studies foregrounded the contextual, experiential, and relational dimensions of engagement. Adaptive timing, personalization, and conversational responsiveness were frequently associated with greater relevance, acceptability, adherence, or perceived support within individual study contexts [14,24,43,44,46,48,50,52,64]. These findings do not demonstrate that any single mechanism independently produced the reported outcome because most interventions combined several components. Gamified and narrative interventions also produced variable patterns across practice frequency, usage, immersion, affective outcomes, and longer-term symptoms [51,55,59,65].

**Table 4.** Engagement-supportive mechanisms, key findings, and design implications.

Study	Primary engagement-supportive mechanism(s)	Key findings (feature focus)	DMHI^a^ design implications
Weintraub et al [12]	Static user-tailored personalization CBT^b^-based self-guided support	App-enhanced intervention lacked effective engagement features; parents showed lower practice than standard CBT	Tailor digital features (reminders and gamification) to engagement needs of adolescents and their caregivers
Sirbu et al [13]	Static user-tailored personalization Self-management support	Daily cognitive techniques (PsyPills) with personalization improved acceptability and reduced stress	Brief structured interventions with personalized content enhance engagement
Wang et al [54]	Dynamic adaptation Conversational/LLM^c^ prompt support	Chain-of-thought prompts generated empathetic, context-aware questions	Use adaptive LLM prompts for personalized dialogue
Heinz et al [24]	Conversational support Emotional responsiveness Adaptive dialogue interaction	Generative AI chatbot provided real-time, empathic interactions	Conversational AI can deliver personalized JITAI^d^-like support
Guo et al [25]	Conversational support Reflective self-monitoring	Adaptive LLM chains improved reliability and safety of mental health dialogues	Implement multistage LLM frameworks for safe, adaptive conversations
Sadprasid et al [55]	Gamified behavioral reinforcement Breathing practice support	Gamified idle breathing increased practice frequency	Gamification can incentivize self-regulation practice
Neupane et al [56]	Context-aware adaptive prompting Biometric-responsive adaptation Conversational support	Wearable-triggered prompts and LLM chatbots created context-aware support	Combine biometric triggers with chatbots for JITAI
Alvarez-Jimenez et al [8]	Social/community support Blended therapeutic engagement	Blended self-help tools and social networking supported varied engagement	Integrate multiple engagement strategies and clinician monitoring
Miller et al [9]	Co-design participation User-centered interaction design	User engagement shaped by co-design processes	Engagement features should be co-designed with users
Dardas et al [57]	Static user-tailored personalization Culturally adaptive support Motivational engagement	Culturally adapted content and motivational interviewing increased adherence	Cultural tailoring and personalization support engagement
Rutherford et al [58]	Flexible delivery Social motivation Experiential engagement	Home-based flexibility vs center-based social motivation	Blend flexibility with social engagement features
Schwarz et al [59]	Narrative immersion Gamified behavioral reinforcement Motivational tailoring	Dynamic tailoring increased narrative presence but did not improve measured immersion; play time and session completion were lower	Assess narrative presence, immersion, and behavioral use separately, alongside sensing burden and usability
Slade et al [60]	User control over sensing Low-burden data collection	User fatigue from frequent sensing; valued autonomy	Allow users to control biometric data collection
Valentine et al [14]	Dynamic adaptive personalization Emotional responsiveness	Participants described check-ins as supporting reflection and self-awareness; low mood and feeling “stuck” hindered use	Allow customizable prompts and self-guided use; address low mood and motivation barriers
Campellone et al [26]	Conversational support Emotional responsiveness Psychoeducational interaction	Generative responses achieved higher rates of user-confirmed accurate reflections (98% vs 69%); satisfaction and bond ratings were similar	Generative AI requires safety guardrails to balance empathy and accuracy
Tong et al [61]	Conversational support Reflective dialogue	The chatbot program improved short-term self-care intentions and mental health literacy; sustained comparative benefits were limited	Offer modular content tailored to user needs
Ying et al [62]	Computational adaptive assessment modeling Personalized mental health state evaluation	Proposed fuzzy logic assessment framework; no engagement features studied	Back-end assessment models should inform personalized interventions
de Graaff et al [63]	Static tailoring Guided conversational support Culturally adapted/social support	Culturally adapted STARS^e^ was feasible and acceptable; lesson and support call completion remained limited	Combine guidance with culturally sensitive content
Coppens et al [64]	Context-aware adaptive prompting Personalized behavioral support	Contextual bandit recommendations increased message relevance and motivation	Use adaptive recommender systems to personalize prompts
Siddals et al [27]	Conversational support Emotional responsiveness Relational engagement	Users appreciated empathetic AI but questioned accuracy	Transparent AI explanations help manage expectations
Wang and Fang [65]	Gamified behavioral reinforcement Team-based social motivation	Team-based gamification boosted affect early, but effects waned	Introduce periodic social challenges to maintain engagement
Aqajari et al [37]	RL^f^ optimization Dynamic adaptive personalization Biometric-responsive adaptation	RL reduced prompts while maintaining detection accuracy	Leverage RL to optimize biometric-triggered JITAI
Kor et al [43]	Static and adaptive JITAI timing Receptivity-informed prompting	In 120 caregivers, retention was 100%; 92.5% found the app easy to use, and 81.7% found it helpful; stress, depression, and caregiver burden decreased from preintervention to postintervention, without a control group	Receptivity models can reduce timing mismatch, but comparative trials are needed before attributing outcome change to adaptation
Elmer et al [44]	Personalized and need-triggered social-support JITAI	EMA^g^ completion was 85.37%; prompts triggered by stated support need were rated as better timed and more helpful than distress threshold triggers; engagement was moderate and heterogeneous	Trigger rules should incorporate expressed support need and practical barriers and not distress thresholds alone
Newby et al [45]	AI-enabled response-adaptive trial allocation	A contextual bandit allocated 1282 students to brief interventions by distress severity; physical activity, mindfulness, and sleep hygiene effects differed across severity strata; engagement and usability were secondary outcomes	Separate adaptation of trial allocation from adaptation of intervention delivery when interpreting engagement mechanisms
Shoshani et al [46]	Conversational AI Therapeutic alliance Dynamic dialogue	In a 995-participant RCT^h^, the AI intervention improved several symptom and well-being outcomes; perceived alliance was associated with engagement (β=.31); engagement was associated with symptom change (β=−.58), with an indirect alliance-to-symptom association through engagement (β=−.18)	Alliance, warmth, and competence should be measured alongside usage; developer conflicts and long-term safety require scrutiny
Valentine et al [47]	Relational and social engagement Practical utility	Among 36 young people, validation, normalization, belonging, and applying therapeutic insights in daily life were associated with sustained platform engagement	Design for relational value and low-effort practical use; address overload, low motivation, and limited reminder support
Dai et al [48]	Context-aware JITAI Emotion recognition Biometric-responsive adaptation	In a 6-week RCT of 120 office workers, adaptive delivery produced higher adherence than fixed scheduling (78% vs 52%) and greater stress reduction; context congruence partly mediated effects	Validate sensing accuracy and decision rules while minimizing burden and protecting privacy
Chow et al [50]	Early-use monitoring Adaptive escalation to human coaching	In a SMART^i^ of 313 breast cancer survivors, the digital treatment improved anxiety but not depression relative to psychoeducation; among participants with low use in week 1, coaching increased the number of days of app use	Use early behavioral signals to target scarce human support, and distinguish engagement effects from symptom effects
Barry et al [51]	Co-designed game Narrative decision-making Peer scenarios	Among 532 adolescents, the 6-week game reduced depressive symptoms relative to an active control over 12 months (adjusted difference at 12 months −1.12, 95% CI −1.85 to −0.38) but did not improve anxiety; beliefs about psychological services improved at 6 weeks	Gamified narrative may support durable benefit when embedded in co-design and tested against an active control; mechanism-specific engagement data remain important
Rouvere et al [52]	Static tailoring of messages and resources	In a large real-world SMART, extra tailoring questions increased disengagement (OR^j^ 1.14); demographic tailoring reduced disengagement (OR 0.90) and increased featured-resource clicks (OR 1.90), whereas need or next-step tailoring did not reduce disengagement	Account for the interaction cost of collecting tailoring data; validate each tailoring variable rather than assuming that greater personalization is better
Xu et al [53]	Conversational relationship Flexible input Privacy and nonjudgmental support	In a 4-week diary study of 26 adults using Woebot and Wysa, 18 reported a bond or light bond; 6 themes linked bonding to conversational control, input fit, caring responses, useful advice, colloquial language, and a private nonjudgmental space	Design for preference fit and relational continuity; offer flexible input and test alliance without equating anthropomorphic rapport with clinical safety

a DMHI: digital mental health intervention.

b CBT: cognitive behavioral therapy.

c LLM: large language model.

d JITAI: just-in-time adaptive intervention.

e STARS: Scalable Technology for Adolescents and Youth to Reduce Stress.

f RL: reinforcement learning.

g EMA: ecological momentary assessment.

h RCT: randomized controlled trial.

i SMART: sequential multiple assignment randomized trial.

j OR: odds ratio.

Table 5 focuses specifically on AI-enabled systems and methodological contributions. It distinguishes studies in which AI functioned as enabling infrastructure (eg, assessment modeling, recommendation, reinforcement learning, response-adaptive allocation, or simulation) from studies in which conversational or adaptive systems directly shaped users’ experiences through dialogue, contextual delivery, emotional support, or personalized interaction. The table also shows how controlled, qualitative, implementation, and computational methods answered different questions concerning efficacy, feasibility, acceptability, safety, relational experience, system logic, and optimization.

Taken together, Tables 4 and 5 indicate that AI was most directly relevant to engagement when embedded within a broader engagement-supportive process than when presented as a standalone feature. They should not be interpreted as rankings of effectiveness because the studies used heterogeneous designs, outcomes, and observation periods and because most did not isolate the effects of individual mechanisms.

**Table 5.** AI-enabled engagement-supportive systems, methodological contributions, and implications for adaptive DMHI^a^ design.

Study	AI component	Key findings (AI focus)	Methodological contributions	Implications for research
Weintraub et al [12]	No AI	No AI component was used; engagement difficulties were reported in the app-enhanced condition	Compared app-enhanced vs standard CBT^b^ and revealed differential engagement	Mixed methods clarify which features support parental engagement
Sirbu et al [13]	No AI	PsyPills used structured REBT^c^ principles without AI	Measured symptom changes and acceptability in a brief intervention	Blinded trials test the efficacy of structured digital interventions
Wang et al [54]	Simulation of LLM^d^ prompts	Chain-of-thought LLM prompts produced empathetic questioning	Evaluated LLM prompting configurations without end user data	Simulation guides design choices before deployment
Heinz et al [24]	Generative AI chatbot	AI chatbot users experienced greater mental health improvements and high completion	Measured mental health outcomes and adherence to chatbot intervention	Controlled trials provide evidence for AI chatbot efficacy but need long-term follow-up
Guo et al [25]	LLM survey	Adaptive LLM chains improved reliability in mental health support	Combined descriptive app survey with architectural development and benchmarking	Surveys inform system design and highlight safety concerns
Sadprasid et al [55]	No AI	Gamified breathing interaction increased short-term engagement and practice frequency through repetitive behavioral reinforcement	Randomized comparison and qualitative feedback captured gamification effects	Mixed methods link usage data with user perceptions of gamification
Neupane et al [56]	Wearable and LLM	Wearable-triggered prompts and LLM chatbots offered contextual support but lacked depth	Reflective narratives illuminated lived experiences of wearable-triggered interventions	Autoethnography uncovers contextual and emotional dimensions often missed by trials
Alvarez-Jimenez et al [8]	No AI	Engagement reliant on blended human-digital support	Real-world evaluation with descriptive statistics and surveys	Implementation research highlights scalability and service integration challenges
Miller et al [9]	No AI	Engagement shaped by co-design and not AI	Analyzed co-design processes and power dynamics	Qualitative inquiry elucidates barriers to participatory engagement
Dardas et al [57]	No AI	Cultural adaptation and motivational interviewing drove engagement	Combined quantitative outcomes with user feedback on culturally adapted content	Mixed methods ensure that cultural tailoring is evaluated for efficacy and acceptability
Rutherford et al [58]	No AI	Flexibility and social motivation were key	Semistructured interviews captured experiences of home vs center programs	Qualitative methods reveal experiential factors impacting engagement
Schwarz et al [59]	No AI	Dynamic tailoring increased narrative presence but did not improve measured immersion; play time and session completion were lower	Tested dynamic vs static exergames across schools	Cluster trials evaluate gamified DHIs^e^ in real-world settings
Slade et al [60]	No AI	User control over sensing increased trust	Linked sensor compliance data with thematic interviews	Integrating quantitative sensing and qualitative insights guides user-centered data collection
Valentine et al [14]	No AI	Users valued self-guided flexibility and reflective check-ins; low mood and motivation could hinder use	Provided insights into user experiences with a transdiagnostic app	Qualitative evaluations refine personalized content and support features
Campellone et al [26]	Generative AI vs rule-based	Generative responses achieved higher rates of user-confirmed accurate reflections (98% vs 69%); satisfaction and bond ratings were similar	Compared generative vs rule-based chatbots on reflection accuracy, satisfaction, bond, and safety	Early-phase trials evaluate AI safety and user satisfaction before scaling
Tong et al [61]	Rule-based chatbots	The chatbot program improved short-term self-care intentions and mental health literacy; sustained comparative benefits were limited	A 2-arm RCT^f^ compared a 10-topic chatbot program with a waitlist control	Factorial RCTs could assess the relative benefit of different modules
Ying et al [62]	Modeling	Computational assessment framework demonstrated how adaptive modeling architectures may support future personalized intervention systems, although no direct engagement outcomes were evaluated	Used fuzzy logic and multicriteria decision-making to build assessment model	Model-based studies lay the groundwork for adaptive systems but need empirical validation
de Graaff et al [63]	Guided chatbot	Culturally adapted STARS^g^ was feasible and acceptable; lesson and support call completion remained limited	Assessed acceptability and retention of a guided chatbot in Jordan	Feasibility trials evaluate cultural adaptations and inform larger RCTs
Coppens et al [64]	Recommender system	Adaptive recommendation logic increased perceived relevance and motivational responsiveness of intervention prompts	Multiweek study compared recommender algorithms	Longitudinal trials gauge sustained engagement and algorithm performance
Siddals et al [27]	Generative AI experiences	Users valued empathic AI but raised concerns about accuracy	Interviews captured user experiences with generative AI chatbots	Qualitative feedback informs ethical guidelines and user education
Wang and Fang [65]	No AI (gamification)	Team-based gamification improved affect without AI	Microrandomized design assessed immediate affective responses to gamified competition	Microrandomized trials identify short-term effects and inform optimal timing^h^
Aqajari et al [37]	RL^i^	Adaptive RL architecture reduced prompting burden while maintaining contextual responsiveness	Offline simulation and online deployment evaluated RL for stress monitoring	Iterative evaluation demonstrates RL applicability in DHIs
Kor et al [43]	Machine learning receptivity models	Static, random, and participant-updated adaptive models selected prompt timing	Within-person delivery phases linked accumulated receptivity data to timing decisions	Compare adaptive and nonadaptive schedules in controlled designs and report model calibration
Elmer et al [44]	Personalized threshold and need trigger logic	Support need triggers were more acceptable than distress variable thresholds	A microrandomized design compared 4 trigger strategies using EMA^j^ and qualitative data	Model practical opportunity and user-declared need and not distress alone
Newby et al [45]	Contextual multiarmed bandit	The algorithm updated allocation probabilities across sequential minitrials by distress severity	Response-adaptive allocation improved trial efficiency but reduced power for some contrasts	Report clearly whether AI adapts research allocation, intervention delivery, or both
Shoshani et al [46]	Conversational AI agent	Therapeutic alliance was associated with engagement and symptom improvement	A 3-arm RCT compared AI, group therapy, and waiting list with 3-month follow-up	Replicate independently and examine safety, alliance durability, and developer conflicts
Valentine et al [47]	No AI	Sustained use was associated with relational experience and practical application	Usage data were integrated with interviews comparing high and low engagers	Mixed methods can explain why similar usage levels may reflect different experiences
Dai et al [48]	Multimodal emotion recognition and context-aware decision rules	Adaptive microinterventions improved adherence relative to fixed scheduling	Delphi development, prototype testing, and a 2-arm RCT linked design decisions to outcomes	Externally validate sensing and mediation results before wider workplace deployment
Chow et al [50]	No AI; SMART^k^ decision rule using week 1 app use	Human coaching increased the number of days of app use among early low engagers	Sequential randomization tested an adaptive support pathway within an effectiveness trial	Predefine decision points and thresholds and report both engagement and clinical outcomes
Barry et al [51]	No AI	The co-designed narrative game produced a small long-term reduction in depressive symptoms but no anxiety effect	An active control and 12-month follow-up strengthened inference about durability	Measure which game and narrative interactions drive engagement and benefit
Rouvere et al [52]	No AI; rule-based tailoring	Tailoring effects depended on the information used, and additional questions themselves increased disengagement	A large real-world sequential experiment separated data collection burden from tailoring effects	Evaluate the net engagement benefit of personalization after accounting for interaction burden
Xu et al [53]	Commercial conversational agents; AI component not experimentally isolated	Relational engagement depended on fit between user preferences and conversational style, input mode, and perceived support	Longitudinal diaries, screenshots, and interviews captured alliance formation over time	Combine behavioral telemetry with repeated qualitative and alliance measures

a DMHI: digital mental health intervention.

b CBT: cognitive behavioral therapy.

c REBT: rational emotive behavior therapy.

d LLM: large language model.

e DHI: digital health intervention.

f RCT: randomized controlled trial.

g STARS: Scalable Technology for Adolescents and Youth to Reduce Stress.

h Microrandomized trials function as methodological frameworks for evaluating adaptive intervention delivery rather than engagement features in themselves.

i RL: reinforcement learning.

j EMA: ecological momentary assessment.

k SMART: sequential multiple assignment randomized trial.

## Discussion

### Principal Findings

This narrative review synthesized engagement-supportive mechanisms across 32 heterogeneous studies [8,9,12-14,24-27,37,43-48,50-65]. Dynamic adaptation, conversational interaction, static tailoring, and social or co-design support were the most frequently represented mechanisms and were commonly combined with contextual or sensing components, although this pattern partly reflects the search focus.

Beyond these frequencies, 3 broader patterns concerned the fit between users’ needs, the system’s decisions, and the support delivered. Firstly, engagement-supportive mechanisms generally operated as layered systems in which sensing or user input informed a decision process and support was delivered through conversational, social, gamified, or other interfaces. Secondly, personalization separated into relatively static tailoring based on predefined characteristics and dynamic adaptation responsive to changing user information or context. Lastly, engagement was represented through behavioral, experiential, relational, and contextual evidence, alongside contributions to system optimization.

### Interpretation and Comparison With Existing Literature

Across the context-aware and JITAI studies, the findings suggest that adaptive decision rules should account for users’ needs and receptivity rather than simply increasing the frequency or complexity of prompting. Adaptive recommendation, reinforcement learning, and wearable-triggered conversational systems illustrated potential to improve message relevance and reduce prompting burden, alongside limitations involving contextual interpretation and conversational depth [37,56,64]. Receptivity-informed timing and context-aware delivery were associated with acceptability or adherence [43,48], while prompts triggered by a stated support need were rated as better timed and more helpful than distress-triggered prompts [44]. However, the quasi-experimental timing study did not isolate the contribution of the timing strategy [43]. Considered together with the wider JITAI literature [29-31,66], these findings suggest that decision rules must represent both vulnerability and opportunity: a detectable stress state does not necessarily mean that a person is able or willing to act.

Biometric responsiveness was less common and generally functioned as enabling infrastructure rather than as a standalone engagement strategy. Physiological, behavioral, or affective signals contributed to stress detection, contextual timing, or adaptive delivery [37,48,56,59], but frequent mobile sensing could create fatigue, and users valued control over data collection [60]. Its potential to make support more contextually relevant while reducing unnecessary prompts must therefore be balanced against device and monitoring burden, privacy, and inference accuracy.

Personalization showed potential benefits alongside interaction costs. Tailored, culturally adapted, and guided interventions were associated with greater perceived relevance or acceptability, reduced stress, or improved emotional regulation [13,14,57,63,64], although digital augmentation did not consistently overcome engagement difficulties [12]. Demographic tailoring reduced disengagement, whereas tailoring based on perceived need or intended next steps did not, and collecting extra tailoring data increased disengagement [52]. Complementary evidence outside the stress-focused corpus showed that interface optimization and reminders improved engagement, whereas adaptive human support improved satisfaction but not engagement [49]. Collectively, these findings suggest that the value of personalization depends on both the information used and the effort required to provide it, qualifying the assumption that more complex adaptation necessarily outperforms simpler improvements in interface design, guidance, or reminders.

Conversational systems provided another pathway to engagement by creating responsive and potentially relational forms of interaction. Alongside favorable acceptability or mental health outcomes [24,63], higher rates of user-confirmed accurate reflections with generative responses [26] and short-term gains in self-care intentions and mental health literacy with topic-based chatbots [61] support assessing conversational responsiveness and self-care learning separately. User experience and alliance findings linked relational value to immediacy and availability [27]; conversational control; input suited to users’ preferred expression; caring responses; and a private, nonjudgmental setting [53], alongside cultural adaptation and human guidance [63]. Perceived alliance was associated with engagement, including an indirect path through engagement to symptom change [46]. These associative and qualitative findings do not establish a causal relational mechanism, and concerns about accuracy and authenticity [27] caution against equating rapport with clinical reliability or safety.

Relational engagement was not limited to interaction with chatbots. Clinical or peer support, social motivation, validation, normalization, belonging, and the practical application of therapeutic insights contributed to engagement [8,47,63,65]. Home-based delivery offered flexibility, whereas center-based programs provided social motivation [58], suggesting that delivery choices should account for practical circumstances and preferences for social interaction. Limited support availability and time constraints [44], cognitive overload and low motivation [47], and increased number of days of app use following targeted coaching among participants with low initial use [50] suggest that human support should address the particular barriers preventing participation. Relational issues also arose during intervention development: co-design revealed how power and competing priorities influenced whose perspectives informed the intervention [9].

Gamification produced variable findings depending on the form and purpose of the game elements. Gamified breathing increased practice frequency [55], dynamic tailoring increased narrative presence without improving measured immersion, while play time and completed sessions were lower in the tailored condition [59], and team competition produced early affective benefits that weakened over time [65]. A co-designed narrative game was associated with lower depressive symptoms than an active control at 12 months, but anxiety did not improve, and engagement quality or narrative resonance were not measured [51]. Considered alongside work on motivationally aligned gamification [19,20], these contrasts indicate that practice, narrative presence, immersion, and affect require separate assessment and that rewards or greater usage alone do not establish meaningful or sustained engagement [7]. The limited narrative evidence [51,59] does not yet establish whether immersion produces sustained use or whether immersion or sustained use mediates mental health outcomes.

AI-related evidence likewise differed in function and maturity. LLM prompting, architectural benchmarking, fuzzy logic assessment, and reinforcement learning studies contributed evidence about system design, assessment, and optimization [25,37,54,62] but did not necessarily demonstrate sustained engagement in everyday use. Response-adaptive allocation concerned research allocation across participants and not within-person adaptation of the standardized interventions they received [45]. The corpus consequently provides evidence of feasibility and experiential potential but limited evidence that AI itself independently improves sustained engagement. AI should therefore be understood according to its functional role within the intervention or research design rather than being treated as a single engagement mechanism.

These findings align with broader digital health and HCI literature showing that engagement cannot be reduced to usage metrics alone [2,5-7,33-36]. Behavioral activity, emotional investment, perceived usefulness, and relational value may diverge across users and contexts. Frameworks emphasize interactions among motivational, immersive, and social processes [7,38-40], but the corpus provided limited evidence on which combinations support meaningful or sustained engagement. Emotional safety, therapeutic credibility, burden, privacy, and contextual inference [28,67,68] may help explain why advances in sensing, adaptive delivery, and AI-enabled support have not been matched by evaluation of users’ experiences and continued participation over time.

### SDT as an Interpretive Lens

SDT helps connect these mechanism-level findings without treating technology as intrinsically motivating. Autonomy may be supported when culturally or individually tailored content increases relevance, users retain meaningful control, and prompts fit their preferences or circumstances [12-14,43,44,52,57,64]. It may be undermined when additional questions, opaque inference, excessive sensing, or poorly timed notifications increase burden [43,44,52,60]. Competence may be supported through achievable practice, feedback, graduated challenge, and opportunities to apply skills, including well-integrated gamified or narrative experiences [51,55,59,65]. Relatedness is reflected in conversational alliance, nonjudgmental support, belonging, co-design, blended care, and selective human coaching [8,9,24,26,27,44,46,47,50,53,58,63]. These are theory-informed alignments, not evidence that SDT was directly tested across the corpus. Their explanatory value lies in distinguishing mechanisms that merely increase exposure from designs that may support volitional, competent, and socially connected participation.

### Implications for Design and Evaluation

Designers should specify what information an adaptive system collects, how that information affects its decisions, and how the resulting support is delivered. Receptivity and declared support needs should be evaluated alongside stress detection, and sensing and tailoring burden should be minimized. Users should be able to understand, control, or contest consequential recommendations. For conversational systems, this also requires attention to accuracy, privacy, explainability, perceived authenticity, emotional dependency, and safe escalation to human support [22,23,26,27,33,34,46,53]. Independent replication is important where intervention developers or commercially affiliated researchers participate in evaluations.

Evaluation should combine behavioral telemetry with experiential, relational, and implementation outcomes matched to the intended engagement pathway. Relevant outcomes include alliance in conversational systems, belonging, validation, and practical use in social platforms and repetition, competence, immersion, or social motivation in gamified systems. Mixed methods designs can connect use or sensing compliance with users’ accounts of burden, relevance, and perceived support [44,47,55,57,60,69]. Qualitative, participatory, autoethnographic, and duoethnographic approaches can reveal power, emotional friction, cultural fit, and trust that usage data may not capture [9,14,27,56,58,70-72]. Future studies should measure psychological need support and motivational quality explicitly if SDT is presented as a design rationale.

Controlled and longitudinal studies can estimate behavioral and clinical effects, with microrandomized trials examining proximal timing effects and factorial trials testing component effects. Sequential multiple assignment randomized trials address adaptive escalation, while response-adaptive designs concern allocation efficiency. Multiphase evaluation can connect modeling, feasibility testing, and controlled comparison with real-world implementation [73], where Proctor taxonomy and the RE-AIM (Reach, Effectiveness, Adoption, Implementation, and Maintenance) framework address acceptability, appropriateness, feasibility, adoption, and maintenance [74,75]. Consistent reporting of engagement definitions, intervention exposure, and adaptive decision rules, together with longer follow-up in routine settings, would improve comparison without reducing engagement to a single universal metric.

### Limitations

This is a narrative review informed by structured procedures and not an exhaustive systematic review or meta-analysis. The searches were restricted to English-language records and selected databases, and the mechanism-focused terminology may have missed relevant interventions. Excluding app store–only evaluations, commercial deployments, and other gray literature may also underrepresent engagement practices used in widely adopted consumer DMHIs [2,11].

Study designs, populations, and engagement measures were heterogeneous, and several interventions combined mechanisms. Mechanism counts should therefore not be interpreted as comparative effectiveness estimates. The narrative synthesis also does not establish that engagement mechanisms caused the reported behavioral or clinical outcomes when those mechanisms were not experimentally isolated.

Most intervention periods were brief; only 10 studies reported assessment beyond the immediate intervention or primary postassessment period, and the evidence remains concentrated in high-income settings. These limitations constrain conclusions about sustained engagement and generalizability. Although the studies from Jordan [57,63] contribute important culturally adapted evidence, the limited representation of lower-resource settings means that assumptions about connectivity, digital literacy, intervention burden, social support, and culturally appropriate engagement cannot be generalized confidently.

Two technical preprints were retained as narrow exceptions because of their emerging architectural contributions [25,37], but they were not treated as clinical effectiveness evidence. The insomnia trial [49] and 4 reviews and meta-analyses [33-36] were used only as external support and were not included in the study tables, mechanism counts, or denominators.

### Conclusions

Engagement in stress-focused DMHIs can be understood as the fit among users’ needs and opportunities, the system’s sensing and decision logic, and the way support is delivered. Adaptive timing, conversational interaction, relational support, motivational design, and meaningful tailoring can contribute to that fit, but none should be assumed effective merely because it is technically sophisticated. The distinction between static tailoring and dynamic adaptation and between behavioral use and experiential or relational engagement should remain explicit in both design and evaluation.

The broader requirement is to build systems that are useful without being intrusive, responsive without being opaque, and scalable without weakening human oversight. Inclusive co-design, independent evaluation, and longitudinal mixed methods evidence remain priorities for translating technological promise into meaningful, sustained, and ethically responsible engagement in stress-management DMHIs.

## Supplementary material

10.2196/90855 Multimedia Appendix 1 Database search strategies and research question operationalization.

10.2196/90855 Checklist 1 PRISMA-P checklist.

## Abbreviations

CBT: cognitive behavioral therapy
DHI: digital health intervention
DMHI: digital mental health intervention
EMA: ecological momentary assessment
HCI: human-computer interaction
JITAI: just-in-time adaptive intervention
LLM: large language model
PRISMA-P: Preferred Reporting Items for Systematic Review and Meta-Analysis Protocols
RCT: randomized controlled trial
RE-AIM: Reach, Effectiveness, Adoption, Implementation, Maintenance
SDT: self-determination theory
