# Supplementary material for: Engagement-Supportive Mechanisms in Stress-Focused Digital Mental Health Interventions With Adaptive, Conversational, and Context-Aware Systems: Narrative Review
Source: JMIR Hum Factors. 2026 Nov 2;13:e90855. doi: 10.2196/90855 (PMC13726798; doi:10.2196/90855)
Supplement: Supplementary file 1 [file humanfactors-v13-e90855-s001.docx]

## **Multimedia Appendix 1. Database Search Strategies and Review Domain Operationalisation**

### **Supplementary Appendix A1. Search strategy overview**

The original searches were conducted in PubMed, Scopus and IEEE Xplore for primarily peer-reviewed English-language records published from January 2018 through April 2025. A focused update retained the same concept blocks and covered May 2025, through August 2026. Six peer-reviewed studies from that focused update met the unchanged criteria [59-64]. A supplementary journal search identified five empirical candidates [67-71]; four met the unchanged criteria and entered the corpus [68-71], while the insomnia study [67] was used only as external component evidence. Four reviews and meta-analyses informed interpretation but were not counted [72-75]. Two technical preprints from the original corpus were retained as a narrow exception [24,41]; no new preprints were included.

### **Supplementary Appendix A2. Review domain operationalisation**

During screening and synthesis, studies were considered relevant if they addressed at least one review domain:. engagement-supportive mechanisms and outcomes; personalisation, biometric feedback, JITAI timing or gamification; AI-enabled adaptive personalisation; or methodological contributions to engagement-informed design. These domains guided interpretation but were not cumulative eligibility requirements.

### **Supplementary Appendix A3. Database search strings**

*PubMed*

(("digital mental health"[Title/Abstract] OR "mental health app"[Title/Abstract] OR "mental health apps"[Title/Abstract] OR mHealth[Title/Abstract] OR eHealth[Title/Abstract] OR "mobile health"[Title/Abstract] OR "digital intervention"[Title/Abstract] OR "digital interventions"[Title/Abstract] OR "digital health intervention"[Title/Abstract] OR "digital health interventions"[Title/Abstract] OR "smartphone app"[Title/Abstract] OR "smartphone apps"[Title/Abstract] OR chatbot*[Title/Abstract] OR "conversational agent"[Title/Abstract] OR "conversational agents"[Title/Abstract] OR "large language model"[Title/Abstract] OR "large language models"[Title/Abstract] OR LLM[Title/Abstract] OR "artificial intelligence"[Title/Abstract] OR "machine learning"[Title/Abstract] OR wearable*[Title/Abstract])

AND

(stress[Title/Abstract] OR distress[Title/Abstract] OR anxiety[Title/Abstract] OR wellbeing[Title/Abstract] OR "well-being"[Title/Abstract] OR "mental wellbeing"[Title/Abstract] OR "mental well-being"[Title/Abstract])

AND

(engagement[Title/Abstract] OR adherence[Title/Abstract] OR retention[Title/Abstract] OR uptake[Title/Abstract] OR participation[Title/Abstract] OR immersion[Title/Abstract] OR motivation[Title/Abstract] OR "user experience"[Title/Abstract] OR usability[Title/Abstract] OR acceptability[Title/Abstract] OR receptivity[Title/Abstract])

AND

(personali*[Title/Abstract] OR tailor*[Title/Abstract] OR adapt*[Title/Abstract] OR "just-in-time"[Title/Abstract] OR JITAI[Title/Abstract] OR "ecological momentary assessment"[Title/Abstract] OR EMA[Title/Abstract] OR biometric*[Title/Abstract] OR gamif*[Title/Abstract] OR "game-based"[Title/Abstract] OR chatbot*[Title/Abstract] OR "conversational AI"[Title/Abstract] OR "large language model"[Title/Abstract] OR "large language models"[Title/Abstract] OR LLM[Title/Abstract] OR "reinforcement learning"[Title/Abstract] OR "context-aware"[Title/Abstract] OR "context aware"[Title/Abstract]))

AND

Original search: ("2018/01/01"[dp] : "2025/04/30"[dp]); focused update: ("2025/05/01"[dp] : "2026/08/26"[dp])

*Scopus*

("digital mental health" OR "mental health app*" OR mhealth OR ehealth OR "mobile health" OR "digital intervention*" OR "digital health intervention*" OR "smartphone app*" OR chatbot* OR "conversational agent*" OR "large language model*" OR LLM OR "artificial intelligence" OR "machine learning" OR wearable*)

AND

(stress OR distress OR anxiety OR wellbeing OR "well-being" OR "mental wellbeing" OR "mental well-being")

AND

(engagement OR adherence OR retention OR uptake OR participation OR immersion OR motivation OR "user experience" OR usability OR acceptability OR receptivity)

AND

(personali* OR tailor* OR adapt* OR "just-in-time" OR JITAI OR "ecological momentary assessment" OR ema OR biometric* OR gamif* OR "game-based" OR "conversational AI" OR "reinforcement learning" OR "context-aware" OR "context aware")

AND PUBYEAR > 2017 AND PUBYEAR < 2027

*IEEE Xplore*

"digital mental health" OR "mental health app" OR mHealth OR eHealth OR "mobile health" OR "digital intervention" OR "digital health intervention" OR "smartphone app" OR chatbot OR "conversational agent" OR "large language model" OR LLM OR "artificial intelligence" OR "machine learning" OR wearable

AND

stress OR distress OR anxiety OR wellbeing OR "well-being" OR "mental wellbeing" OR "mental well-being"

AND

engagement OR adherence OR retention OR uptake OR participation OR immersion OR motivation OR "user experience" OR usability OR acceptability OR receptivity

AND

personalization OR personalisation OR tailoring OR adaptive OR "just-in-time" OR JITAI OR "ecological momentary assessment" OR EMA OR biometric OR gamification OR "game-based" OR "conversational AI" OR "reinforcement learning" OR "context-aware" OR "context aware"
