# Supplementary material for: Engagement-Supportive Mechanisms in Stress-Focused Digital Mental Health Interventions With Adaptive, Conversational, and Context-Aware Systems: Narrative Review
Source: JMIR Hum Factors. 2026 Nov 2;13:e90855. doi: 10.2196/90855 (PMC13726798; doi:10.2196/90855)
Supplement: Supplementary file 2 [file humanfactors-v13-e90855-s002.docx]

### **PRISMA-P (Preferred Reporting Items for Systematic review and Meta-Analysis Protocols) 2015 checklist: recommended items to address in a systematic review protocol***

### **Engagement-Supportive Mechanisms in Stress-Focused Digital Mental Health Interventions: A Narrative Review of Adaptive, Conversational and Context-Aware Systems**

| Section and topic | Item No | Checklist item | **Author response** |
| --- | --- | --- | --- |
| ADMINISTRATIVE INFORMATION | | | |
| Title: |  |  |  |
| Identification | 1a | Identify the report as a protocol of a systematic review | Not Applicable. Identified as a narrative review. |
| Update | 1b | If the protocol is for an update of a previous systematic review, identify as such | Not Applicable. |
| Registration | 2 | If registered, provide the name of the registry (such as PROSPERO) and registration number | Not Applicable. |
| Authors: |  |  |  |
| Contact | 3a | Provide name, institutional affiliation, e-mail address of all protocol authors; provide physical mailing address of corresponding author | 1. Munesu Maminimini (MM); Department of General Psychology, University of Padua; Digital Health Research, Fondazione Bruno Kessler, Trento, Italy; [mmaminimini@fbk.eu](mailto:mmaminimini@fbk.eu); Via Sommarive, 18, 38123 Povo (Trento)Italy 2. Soffia Asteria Piffer (SAP); Digital Health Research, Fondazione Bruno Kessler, Trento, Italy; [sopiffer@fbk.eu](mailto:sopiffer@fbk.eu) 3. Silvia Gabrielli (SG); Digital Health Research, Fondazione Bruno Kessler, Trento, Italy; [sgabrielli@fbk.eu](mailto:sgabrielli@fbk.eu) |
| Contributions | 3b | Describe contributions of protocol authors and identify the guarantor of the review | 1. MM: Conceptualisation, Methodology, Investigation, Data curation, Formal analysis, Visualization, and Writing – original draft. 2. SAP: Methodology, Investigation, Data curation, Validation,and Writing – review & editing. 3. SG: Conceptualisation, Validation, Supervision, and Writing – review & editing. |
| Amendments | 4 | If the protocol represents an amendment of a previously completed or published protocol, identify as such and list changes; otherwise, state plan for documenting important protocol amendments | Not Applicable |
| Support: |  |  |  |
| Sources | 5a | Indicate sources of financial or other support for the review | Not Applicable |
| Sponsor | 5b | Provide name for the review funder and/or sponsor | Fondazione Bruno Kessler |
| Role of sponsor or funder | 5c | Describe roles of funder(s), sponsor(s), and/or institution(s), if any, in developing the protocol | Not Applicable |
| INTRODUCTION | | | |
| Rationale | 6 | Describe the rationale for the review in the context of what is already known | Engagement remains difficult to sustain in stress-focused digital mental health interventions (DMHIs). Evidence on how engagement mechanisms are implemented, combined and evaluated is fragmented, with inconsistent measures and limited longitudinal evidence. The review addresses these gaps.  Location: Introduction. |
| Objectives | 7 | Provide an explicit statement of the question(s) the review will address with reference to participants, interventions, comparators, and outcomes (PICO) | To synthesise and critically evaluate how stress-focused DMHIs conceptualise and operationalise engagement, how personalisation, biometric feedback, JITAI timing, gamification and AI-enabled approaches support it, and how different methodologies contribute to understanding implementation and effects.  Location: Introduction, final paragraph. |
| METHODS | | | |
| Eligibility criteria | 8 | Specify the study characteristics (such as PICO, study design, setting, time frame) and report characteristics (such as years considered, language, publication status) to be used as criteria for eligibility for the review | Eligible English language reports described digital interventions primarily targeting stress or related affective outcomes in preventative mental health, at least one engagement-supportive mechanism, and empirical, qualitative, implementation, simulation, modelling or system-design evidence. Protocols, reviews, dissertations, non-digital and non-mental health interventions were excluded. Peer review was required, with two specified technical-preprint exceptions. Commercial apps, app store only evaluations and other grey literature were excluded unless evaluated in an eligible peer-reviewed study. Search coverage extended from January 2018 to August 2026.  Location: Methods, Eligibility Criteria and Search Strategy. |
| Information sources | 9 | Describe all intended information sources (such as electronic databases, contact with study authors, trial registers or other grey literature sources) with planned dates of coverage | The original searches covered PubMed, Scopus and IEEE Xplore for January 2018 to April 2025. A focused update covered May 2025 to August 2026 using the same concept blocks, supplemented by a journal search of recent peer-reviewed publications.  Location: Methods, Search Strategy; Multimedia Appendix 1, A1. |
| Search strategy | 10 | Present draft of search strategy to be used for at least one electronic database, including planned limits, such that it could be repeated | Database specific search strings are provided, including PubMed field tags and original and update publication date limits. Search concepts combine digital mental health, stress or related affective outcomes, engagement and adaptive mechanisms.  Location: Multimedia Appendix 1, A3; Methods, Search Strategy; Table 1. |
| Study records: |  |  |  |
| Data management | 11a | Describe the mechanism(s) that will be used to manage records and data throughout the review | Data were extracted using a structured 17-item form and summarised in study-level tables. Duplicate removal and screening counts are reported in the study-selection flow diagram.  Location: Methods, Study Selection and Data Extraction; Results, Study Selection; Figure 1; Tables 2-5. |
| Selection process | 11b | State the process that will be used for selecting studies (such as two independent reviewers) through each phase of the review (that is, screening, eligibility and inclusion in meta-analysis) | Two authors independently screened titles/abstracts and assessed full texts in the original review, with disagreements resolved through discussion with the third author. For the update, the first author screened studies and the third author verified draft eligibility decisions using unchanged criteria.  Location: Methods, Study Selection and Data Extraction; Figure 1. |
| Data collection process | 11c | Describe planned method of extracting data from reports (such as piloting forms, done independently, in duplicate), any processes for obtaining and confirming data from investigators | The first and second authors extracted data from the original studies using the structured 17-item form. For update studies, the first author extracted data using the same form and the third author verified the extracted data and interpretations.  Location: Methods, Study Selection and Data Extraction. |
| Data items | 12 | List and define all variables for which data will be sought (such as PICO items, funding sources), any pre-planned data assumptions and simplifications | The extraction form captured publication details, population, setting, intervention modality, duration, follow-up, study design, engagement definitions and measures, engagement-supportive mechanisms, AI or sensing components, key findings and design implications. Eligibility decisions and mechanism coding were kept separate.  Location: Methods, Study Selection and Data Extraction and Synthesis Approach; Tables 2–5. |
| Outcomes and prioritization | 13 | List and define all outcomes for which data will be sought, including prioritization of main and additional outcomes, with rationale | The synthesis distinguished behavioural engagement, including uptake, adherence, use, retention and completion, from cognitive, emotional, experiential and social or relational engagement, including relevance, motivation, immersion, trust and perceived support. A prespecified primary/secondary outcome hierarchy is not reported.  Location: Introduction; Methods, Synthesis Approach; Tables 4-5. |
| Risk of bias in individual studies | 14 | Describe anticipated methods for assessing risk of bias of individual studies, including whether this will be done at the outcome or study level, or both; state how this information will be used in data synthesis | A formal risk-of-bias assessment is not performed. Study designs, findings and limitations inform the narrative interpretation.  Location: Methods, Synthesis Approach; Discussion, Limitations; Tables 4-5. |
| Data synthesis | 15a | Describe criteria under which study data will be quantitatively synthesised | Narrative synthesis was selected because the evidence spans heterogeneous clinical trials, qualitative investigations, implementation research and computational studies. Quantitative summaries describe study characteristics and mechanism frequencies; pooled intervention effects were not estimated.  Location: Methods, Design and Synthesis Approach. |
|  | 15b | If data are appropriate for quantitative synthesis, describe planned summary measures, methods of handling data and methods of combining data from studies, including any planned exploration of consistency (such as I^2^, Kendall’s τ) | Not applicable. No meta-analysis was conducted. Quantitative summaries consist of descriptive study counts and mechanism frequencies.  Location: Methods, Design and Synthesis Approach. |
|  | 15c | Describe any proposed additional analyses (such as sensitivity or subgroup analyses, meta-regression) | Descriptive analyses examine mechanism co-occurrence and engagement-feature adoption across study designs. Sensitivity analyses, subgroup meta-analyses and meta-regression are not reported.  Location: Results, Mechanism Co-occurrence and Patterns in Engagement Feature Adoption by Methodology; Figure 2; Table 3. |
|  | 15d | If quantitative synthesis is not appropriate, describe the type of summary planned | A two-stage synthesis combines descriptive mapping of study characteristics with narrative interpretation of behavioural, cognitive, experiential, relational and methodological evidence. Overlapping mechanisms were coded in eight categories, and self-determination theory informed the interpretation of motivational and experiential engagement.  Location: Methods, Synthesis Approach. |
| Meta-bias(es) | 16 | Specify any planned assessment of meta-bias(es) (such as publication bias across studies, selective reporting within studies) | A formal assessment of publication bias or selective reporting is not reported. Search restrictions and potential underrepresentation of grey literature are discussed.  Location: Discussion, Limitations. |
| Confidence in cumulative evidence | 17 | Describe how the strength of the body of evidence will be assessed (such as GRADE) | The interpretation considers study designs, heterogeneous populations and measures, intervention duration, follow-up, setting concentration and limits on causal inference. Formal certainty grading using a framework such as GRADE is not reported.  Location: Discussion, Principal Findings and Limitations; Tables 4-5. |

*** It is strongly recommended that this checklist be read in conjunction with the PRISMA-P Explanation and Elaboration (cite when available) for important clarification on the items. Amendments to a review protocol should be tracked and dated. The copyright for PRISMA-P (including checklist) is held by the PRISMA-P Group and is distributed under a Creative Commons Attribution Licence 4.0.**

*From: Shamseer L, Moher D, Clarke M, Ghersi D, Liberati A, Petticrew M, Shekelle P, Stewart L, PRISMA-P Group. Preferred reporting items for systematic review and meta-analysis protocols (PRISMA-P) 2015: elaboration and explanation. BMJ. 2015 Jan 2;349(jan02 1):g7647.*
